# Little information, great impact? A clinical tool for the prediction of electroconvulsive therapy effectiveness in depression

**DOI:** 10.1192/bjo.2026.10977

**Published:** 2026-02-16

**Authors:** Michael Belz, Isabel Methfessel, Matthias Besse, Melvin Heinisch, Wolfgang Strube, Joshua Tritsch, Alkomiet Hasan, David Zilles-Wegner

**Affiliations:** Department of Psychiatry and Psychotherapy, University Medical Center Göttingen, D-Göttingen, Germany; Department of Psychiatry, Psychotherapy and Psychosomatics, Medical Faculty, University of Augsburg, Augsburg, Germany; DZPG (German Center for Mental Health), Partner Site, München/Augsburg, Germany

**Keywords:** Electroconvulsive therapy (ECT), response prediction, treatment outcome, depressive disorder

## Abstract

**Background:**

The effectiveness of electroconvulsive therapy (ECT) for depression strongly depends on patient characteristics. Clinical factors may increase (e.g. age, psychotic symptoms) or decrease (e.g. episode duration) response rates.

**Aims:**

This prospective study aimed to develop an instrument for the prediction of ECT response in patients with unipolar depression.

**Method:**

*N* = 45 patients were assessed using the Göttingen Response to ECT Assessment Tool (GREAT; seven items, 0 to 14 points). Clinical outcome was measured using the Montgomery Åsberg Depression Rating Scale (MADRS). Response was defined as ≥ 50% MADRS-improvement or a clinical global impression improvement (CGI-I) score ≤ 2. Analyses were conducted between responders and non-responders.

**Results:**

Results showed a high correlation between GREAT-score and dichotomous response (*r* = 0.585) as well as MADRS-improvement (*r* = 0.554, both *p* < 0.001). Receiver operating characteristic (ROC)-analysis yielded an area under the curve (AUC) of 0.841 (asymptotic significance: *p* < 0.001). A cut-off point at ≥7 points predicted ECT response in individual cases with 80% accuracy. GLM-analyses showed a significantly better MADRS-improvement for patients with a GREAT-score ≥ 7 *v*. < 7 (interaction-effect: *p* < 0.001).

**Conclusions:**

Our prospective study shows that an instrument consisting of seven clinical items is able to predict ECT response in depression with good accuracy. Limitations include a relatively small sample size and the lack of further potential predictors suggested by recent studies. GREAT will thus be modified to further improve its accuracy. Currently, it may give clinicians a relevant estimate of the likelihood and the extent of the individual response to ECT.

Electroconvulsive therapy (ECT) is the most effective treatment for severe or treatment-resistant depressive disorders. However, there is a current debate on the role of ECT in treatment algorithms for depression, as it might be useful to distinguish particularly severe forms of depressive illness from merely treatment-resistant cases.^1^ This is reflected by markedly different response rates of ECT depending on the specific study populations.^2,3^ While unselected samples show response rates of about two thirds of the patients, rates may rise up to 90% in populations with psychotic depression^4^ and be markedly reduced in patients with previous pharmacological treatment resistance.^5^ These findings have been corroborated in more recent studies.^6,7^ Several systematic reviews and meta-analyses sought to identify clinical factors associated with response to ECT in patients with depression.^5,8,9^ According to these studies, higher age, presence of psychotic symptoms and higher symptom severity were positively associated with ECT response, while longer episode duration and more medication failures showed a negative association. During the last years, further clinical factors were identified that may have a positive (presence of psychomotor symptoms)^10^ or negative (comorbid borderline personality disorder)^11^ impact on ECT response. It is important to note that these factors do not exist independently of each other. For example, some studies suggest that the effect of age is substantially mediated by the presence of psychotic and psychomotor symptoms and a shorter episode duration.^10,12^

Recent studies aimed to develop and test prediction models based on these factors. Although resulting models showed a certain predictive validity, the explained variance^13^ and area under the curve (AUC) values^14^ were rather low and not yet expedient for decision-making in clinical practice. As there is only preliminary evidence for the utility of neuroimaging^15,16^ or molecular markers^17^ to predict ECT response, there is still an unmet need for instruments that are able to guide clinical decisions in psychiatry and facilitate individualised treatment approaches. ECT in particular may be predestined for the development of predictive tools due to its highly standardised application. Furthermore, given the still limited resources and availability of ECT,^18–20^ it is crucial to offer timely treatment to those patients who are more likely to benefit from it and avoid a sometimes burdensome procedure for those who will not.

The aim of our study was to develop and test an instrument for the prediction of ECT response in patients with unipolar major depression. The tool was designed to be easy to administer, time-efficient and allow clinical application independent of the specific setting and availability of more elaborate technical methods. For this purpose, we summarised established clinical factors associated with ECT response that are also readily available in routine clinical assessment in an easy to use scoring instrument (Göttingen Response to ECT Assessment Tool, GREAT). Based on the available meta-analyses and further recent studies,^5,8–11^ we selected the following factors: (1) age, (2) episode duration, (3) insufficient response to pharmacotherapy, (4) depression severity, (5) psychotic symptoms, (6) psychomotor symptoms and (7) borderline personality disorder.

## Method

### Sample

All data reported in this study were collected prospectively. The following inclusion criteria were applied: (a) clinical indication for ECT, (b) age of ≥18 years and (c) moderate to severe unipolar depressive episode (ICD-10: F32.1 to F32.3 and F33.1 to F33.3). Exclusion criteria comprised (a) dementia (ICD-10: F00 to F03), (b) organic affective disorders (ICD-10: F06.3) and (c) current substance abuse (except for tobacco; ICD-10: F10 to F16 and F18 to F19). (d) As the GREAT-score (see below for details) was designed to predict the outcome of ECT, patients who did not complete the ECT series as prescribed/indicated by the treating psychiatrist were excluded.

Patients had either given general consent to the use of research data as part of the treatment contract or gave their written informed consent prior to the study. The authors assert that all procedures contributing to this work comply with the ethical standards of the relevant national and institutional committees on human experimentation and with the Helsinki Declaration of 1975, as revised in 2013. All procedures involving human patients were approved by the Institutional Review Board (IRB) of the University Medical Center Göttingen (ethical vote 14/5/23) and of the LMU/Augsburg Review Board (ethical vote 23-1005).

### ECT treatment

Included patients received ECT treatment from July 2023 to March 2025, with mean 12.24 (s.d. = 2.47) sessions per ECT series. A Thymatron IV device (Somatics, LLC., Lake Bluff, IL, USA) was used. Brief pulse stimulation and the double-dose programme were applied (maximum stimulus dose of 1008 mC, 200%) with age-based dosing in the initial session (stimulus dose = age in right unilateral (RUL), stimulus dose = half age in bilateral stimulation). Initial electrode placement and potential changes in the treatment course were determined according to clinical considerations (symptom severity, early response, tolerability/cognitive side effects) and included bifrontal, RUL, bitemporal and left anterior right temporal (LART) position. Seizure quality was determined by assessing ictal EEG parameters (seizure duration and amplitude). Stimulus dose could be adjusted in case of short (< 20s) or low amplitude seizures. For anesthesia, patients either received a combination of esketamine and propofol (0.7 and 0.5 mg per kg, respectively), methohexital (1 mg per kg) or etomidate (0.2 to 0.3 mg per kg). Succinylcholine (1 mg per kg) was used as muscle relaxant in all cases. All dosages could be adjusted according to clinical requirements.

### Study design

#### GREAT-score

As described in the introduction, our aim was to develop and test a clinical instrument for the prediction of ECT response in patients with depression. To make this instrument easy to use in clinical practice, all necessary information had to be readily available from routine clinical assessment. Seven factors that fulfilled this criterion and that have been associated with response to ECT in meta-analyses and other recently published studies^5,8–11^ were selected and included as one item each in our assessment tool (GREAT): (1) age, (2) episode duration, (3) insufficient response to pharmacotherapy, (4) depression severity, (5) psychotic symptoms, (6) psychomotor symptoms and (7) borderline personality disorder. Please see Table 1 for all translated items. Each item was formulated as short note, e.g. item 2: ‘duration of current episode’ and rated metrically between ‘0’, ‘1’ and ‘2’ (item 2: ‘0’ ≥ 24 months, ‘1’ = 12–23 months, ‘2’ < 12 months). All values were set in such a way that a higher value hypothetically indicated a higher probability of response (e.g. item 2: longer duration of the current episode led to a lower score; see Table 1 for more examples). The GREAT-score was calculated by adding the scores of the seven single items (range: 0 to 14 points). It is important to emphasise that the gradations of the ratings are simplified and to some extent arbitrary. In the absence of clear and uniform definitions or conventions from the literature, we determined the gradations according to clinical judgement and plausibility. Although we partly adopted categories from previous studies and meta-analyses, it is rather unlikely that these gradations already represent the most reliable variant for the prediction of ECT response.

**Table 1:** GREAT (Göttingen Response to ECT Assessment Tool)-score: translated items

Items	Answer options
I1 : Age	**0** = 18–49 **1** = 50–64 **2** ≥ 65
I2 : Duration of current episode	**0** ≥ 24 months **1** = 12–23 months **2** = < 12 months
I3 : Insufficient response to pharmacotherapy in the current episode	**0** = insufficient response to one or more antidepressants plus one or more augmentation strategies (lithium or antipsychotic) **1** = insufficient response to two or more antidepressants **2** = insufficient response to a maximum of one antidepressant
I4 : Depression severity^a^ (MADRS or HRSD17; if not possible CGI-S)	**0** = MADRS ≤ 19 (*or* HRSD17^c^ ≤ 15 *or* CGI-S ≤ 3) **1** = MADRS = 20–34 (*or* HRSD17 = 16–26 *or* CGI-S = 4–5) **2** = MADRS ≥ 35 (*or* HRSD17 ≥ 27 *or* CGI-S = 6–7)
I5 : Psychotic symptoms^a^^,^^b^	**0** = no psychotic symptoms **1** = conspicuous thinking in terms of content and intensity (e.g. marked feelings of guilt, hypochondriac ideas or financial worries), but not delusional **2** = manifest, stable delusions or other clearly psychotic symptoms (e.g. hallucinations)
I6 : Psychomotor symptoms^a^ (agitation or retardation)	**0** = no psychomotor disturbances in movement and speech **1** = mild to moderate or only temporary signs of psychomotor retardation or agitation **2** = marked or continuous signs of psychomotor retardation (slowing, mutism, stupor) or agitation (excitement, tension, anxiety)
I7 : Comorbid borderline PD	**0** = manifest diagnosis of borderline PD **1** = clinical indications of borderline PD based on screening tests, clinical observation, medical history (e.g. marked emotional lability, fluctuation of mood symptoms in the context of interpersonal stress, non-suicidal self-harm) **2** = no signs of borderline PD from diagnostics and clinical observation

MADRS, Montgomery Åsberg Depression Rating; HRSD, Hamilton Rating Scale for Depression; CGI, clinical global impression improvement; PD, personality disorder.

The Göttingen Response to ECT Assessment Tool (GREAT) sum-score can be calculated by adding all values (0 to 14 points).

a. Rating ≤7 days before scoring.

b. Pseudo-hallucinations, dissociative or intrusive symptoms (flashbacks) have to be distinguished and are rated ‘0’.

c. The HRSD17 was not used in this study, but it can be considered as an alternative to the MADRS scale for the external assessment of depression severity.

#### Pre- and post-measurement

The GREAT-score was assessed 1–7 days prior to the first ECT treatment (pre-measurement), along with clinical routine data. To quantify the severity of depressive symptoms, the Montgomery Åsberg Depression Rating Scale (MADRS, 0 to 60 points) was used. At post-measurement, the MADRS was used again, along with the clinical global impression improvement scale (CGI-I, ranging from ‘1’ = *‘*very much improved’ to ‘7’ = *‘*very much worse’). A MADRS-reduction of ≥ 50% was defined as ‘responder’.^21^ If pre- and post-measurements of MADRS were not available, ‘responder’ was defined by CGI-I ≤ 2.^22^

### Statistical analysis

IBM SPSS Statistics 30 for Windows (IBM Corp., Armonk, NY; see https://www.ibm.com/products/spss-statistics) was used for data analysis. For descriptive representation, we computed means and s.d.s for metric variables, along with frequencies for categorical variables and correlations (*r*; metric: Pearson, binary: phi-coefficient).

First, to differentiate the GREAT-score between responders and non-responders, a *t*-test with effect size (Cohen’s *d*) was calculated. Second, we carried out a receiver operating characteristic (ROC)-analysis with response (‘yes’ versus ‘no’) as binary state variable, and the GREAT-score as test variable. Third, we derived a possible cut-off point for the GREAT-score from the ROC-analysis, and compared its discriminatory ability with the responder status as defined *post hoc* by MADRS-improvement. To accomplish that, we created two general linear models for repeated measures (GLM), each with pre- and post-measured MADRS scores as dependent variables. The first GLM was used as reference model: we used responder status as between-groups discriminator (‘yes’ versus ‘no’). Within the second GLM, groups were discriminated by the GREAT-score cut-off (‘below’ and ‘greater than/equal to’). For both GLMs, interaction effects were tested for significance (group × repeated measurement), along with pairwise comparisons between groups at pre- and post-measurement to secure a possible interaction. As we conducted four statistical tests for our primary endpoint (1 × *t*-test, 1 × ROC-analysis (asymptotic significance), 2 × GLM), *p*-values were corrected using the Bonferroni method. Initial significance was set at *p* < 0.05, two-tailed, *p* was then adjusted for four tests (*p*_*adj*_ = *p* × 4). Pairwise comparisons for both GLM were also corrected within each model.

Additionally, we conducted explorative tests, and an explorative analysis for all seven GREAT items, assessing correlations with response and distribution of the categories (‘0’, ‘1’, ‘2’) between responders and non-responders. To assess if observed distributions deviated significantly between both groups, a chi-squared test and six Fisher exact tests (with Freeman–Halton extension for 2 × 3 matrices) were used – see the Results section for details.

## Results

### ECT response and GREAT

All patients were in-patients, including external referrals for ECT. Data on previously received ECT or repetitive transcranial magnetic stimulation (rTMS) was not recorded. A total of 20 patients were excluded from the study. In 18 of these patients, the ECT series was discontinued prematurely, as decided by the treating psychiatrist. Reasons for discontinuation included changes in patient preference, side effects or medical complications. Two further patients were excluded from the final analysis due to an already marked and clinically relevant improvement of depression prior to the beginning of ECT, as reported by the treating psychiatrists. The excluded patients did not differ from included patients at baseline in demographics (gender: *p* = 0.999; age: *p* = 0.480), GREAT-score (*p* = 0.619) and depression severity (MADRS; *p* = 0.188).

A final sample of *N* = 45 patients from the University Medical Centers Göttingen (Department of Psychiatry and Psychotherapy) and Augsburg (Department of Psychiatry, Psychotherapy and Psychosomatics) were included in the study. Included patients were between the ages of 21 and 86 years (mean 56.18, s.d. = 19.99) and female by majority (*n* = 36, 80.0%; male: *n* = 9, 20.0%, non-binary: *n* = 0, 0.0%). Please see Table 2 for sample details and a comparison of responders versus non-responders as well as details on ECT treatment. Out of *N* = 45 patients, *n* = 40 provided a MADRS pre- and post-measurement, which decreased from *M*_*pre*_ = 34.95 (s.d. = 6.41) to *M*_*post*_ = 18.05 (s.d. = 11.16; *t*(39) = 9.33, *p* < 0.001, *d* = 1.48). In sum, response-status of *n* = 40 patients based on MADRS (*n* = 23 responders, *v*. *n* = 17 non-responders), whereas *n* = 5 patients based on CGI-I (*n* = 2 responders, *v*. *n* = 3 non-responders). Overall, ECT treatment led to a response-rate of 55.6% (*n* = 25 out of 45). As a definition of response by CGI-I can be criticised as potentially biased, we also compared the response-definition by CGI-I and MADRS within the *n* = 40 patients for whom both measures were available. A discrepancy was found in *n* = 2 cases (5.0%), in which the status ‘response’ was given according to CGI-I but overruled by the MADRS pre–post difference missing the ≥ 50% criterion (improvement: 34.4% and 45.7%, respectively). A match of both measures was found for the majority (*n* = 38, 95.0%).

**Fig. 1:**
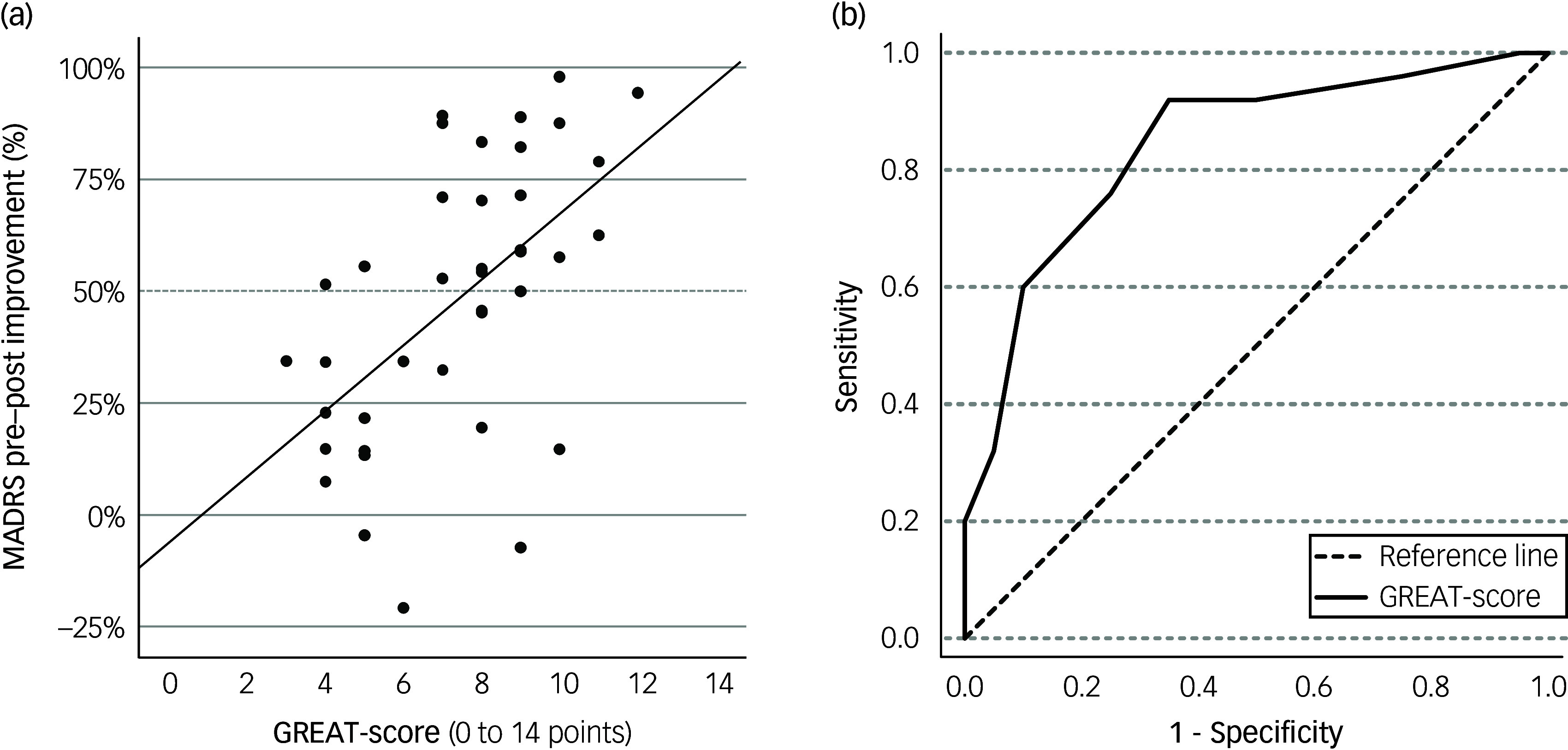
Göttingen Response to ECT Assessment Tool (GREAT): correlation with Montgomery Åsberg Depression Rating Scale (MADRS) improvement and receiver operating characteristic (ROC)-analysis. (a) Scatterplot with regression line for (1) GREAT-score (0 to 14 points) and (2) MADRS-improvement from pre- to post-electroconvulsive therapy (ECT) (percentage; positive values indicate improvement); (b) ROC-curve with reference line for GREAT-score (0 to 14 points; see Table 1 for all items); *N* = 45.

Please see Table 3 for correlations. The GREAT-score correlated significantly with response (*r* = 0.585, *p* < 0.001), MADRS-delta (absolute symptom reduction; *r* = 0.532, *p* < 0.001) and MADRS-improvement (symptom reduction in percent; *r* = 0.554, *p* < 0.001). As illustrated by a scatterplot with regression line (see Fig. 1(a)), each additional point on the GREAT-score led to an improvement of approximately 7.3% on the MADRS (*y* = −0.062 + 0.073 * x), with an explained variance of 30.7% (linear *R* ^2^). In line with these findings, the subgroup of responders had a significantly higher GREAT-score (mean 8.84, s.d. = 2.14) compared to non-responders (mean 5.95, s.d. = 1.91; *t*(43) = 4.73, *p* < 0.001), leading to an effect size of *d* = 1.42.

**Table 2:** Patient characteristics

Variable	Total sample (*N* = 45)	Responder (*n* = 25)	Non-responder (*n* = 20)	*p* ^b^
Age	mean = 56.18 ± 19.99	mean = 65.24 ± 17.03	mean = 44.85 ± 17.77	< 0.001***
Gender (male; female)	9 (20.0%); 36 (80.0%)	3 (12.0%); 22 (88.0%)	6 (30.0%); 14 (70.0%)	= 0.134
GREAT-score	mean = 7.56 ± 2.48	mean = 8.84 ± 2.14	mean = 5.95 ± 1.91	< 0.001***
MADRS Δ^a^	mean = 17.18 ± 11.64	mean = 24.70 ± 8.23	mean = 7.00 ± 6.78	< 0.001***
ECT: Electrode placement				–
Bifrontal	*n* = 11 (24.4%)	*n* = 8 (32.0%)	*n* = 3 (15.0%)	
Right unilateral	*n* = 9 (20.0%)	*n* = 7 (28.0%)	*n* = 2 (10.0%)	
Left anterior, right temporal	*n* = 1 (2.2%)	*n* = 1 (4.0%)	*n* = 0 (0.0%)	
Bitemporal	*n* = 5 (11.1%)	*n* = 1 (4.0%)	*n* = 4 (20.0%)	
Change during series	*n* = 19 (42.2%)	*n* = 8 (32.0%)	*n* = 11 (55.0%)	
Number of ECT-sessions	mean = 12.24 ± 2.47	mean = 12.00 ± 2.40	mean = 12.55 ± 2.58	= 0.464
ECT: duration of series (days)	mean = 31.96 ± 10.49	mean = 31.56 ± 11.98	mean = 32.45 ± 8.54	= 0.781
ECT: stimulation dose (%)	mean = 75.49 ± 39.97	mean = 87.72 ± 43.32	mean = 60.20 ± 29.79	= 0.020*
Main diagnoses (ICD-10)				–
F33.1	*n* = 1 (2.2%)	*n* = 0 (0.0%)	*n* = 1 (5.0%)	
F33.2	*n* = 34 (75.6%)	*n* = 18 (72.0%)	*n* = 16 (80.0%)	
F33.3	*n* = 7 (15.6%)	*n* = 6 (24.0%)	*n* = 1 (5.0%)	
F32.2	*n* = 3 (6.7%)	*n* = 1 (4.0%)	*n* = 2 (10.0%)	
Antidepressant				–
SSRI	*n* = 6 (13.3%)	*n* = 4 (16.0%)	*n* = 2 (10.0%)	
SSNRI	*n* = 17 (37.8%)	*n* = 11 (44.0%)	*n* = 6 (30.0%)	
Tricyclic	*n* = 4 (8.9%)	*n* = 2 (8.0%)	*n* = 2 (10.0%)	
Mirtazapine	*n* = 3 (6.7%)	*n* = 3 (12.0%)	*n* = 0 (0.0%)	
Other	*n* = 4 (8.9%)	*n* = 0 (0.0%)	*n* = 4 (20.0%)	
Combination	*n* = 9 (20.0%)	*n* = 4 (16.0%)	*n* = 5 (25.0%)	
None	*n* = 2 (4.4%)	*n* = 1 (4.0%)	*n* = 1 (5.0%)	
Antipsychotic				–
Atypical	*n* = 14 (31.1%)	*n* = 10 (40.0%)	*n* = 4 (20.0%)	
Low-potency	*n* = 6 (13.3%)	*n* = 2 (8.0%)	*n* = 4 (20.0%)	
High-potency	*n* = 2 (4.4%)	*n* = 2 (8.0%)	*n* = 0 (0.0%)	
Combination	*n* = 11 (24.4%)	*n* = 9 (36.0%)	*n* = 2 (10.0%)	
None	*n* = 12 (26.7%)	*n* = 2 (8.0%)	*n* = 10 (50.0%)	
Mood stabiliser				–
Lithium	*n* = 6 (13.3%)	*n* = 2 (8.0%)	*n* = 4 (20.0%)	
Anticonvulsants	*n* = 4 (8.9%)	*n* = 4 (16.0%)	*n* = 0 (0.0%)	
None	*n* = 35 (77.8%)	*n* = 19 (76.0%)	*n* = 16 (80.0%)	

mean, mean ± s.d.; GREAT, Göttingen Response to ECT Assessment Tool; MADRS, Montgomery Åsberg Depression Rating Scale Δ (pre-measurement minus post-measurement; positive values indicate higher delta); ECT, electroconvulsive therapy; SSRI, selective serotonin reuptake inhibitors; SSNRI, elective serotonin and norepinephrine reuptake inhibitor.

a. A total of *n* = 40 patients provided a pre- and post-measurement of the MADRS (responder: *n* = 23, non-responder: *n* = 17).

b. Pairwise comparisons between responders and non-responders depending on scale level, *p*-values for: multiple *t*-tests, χ^2^ test (gender).

**Table 3:** Correlations

Variable	1	2	3	4	5	6	7	8
GREAT-score	–							
Age (years)	0.619***	–						
Gender (binary)	0.023	0.044	–					
Responder	0.585***	0.513***	0.224	–				
MADRS Δ	0.532***	0.492**	0.160	0.761***	–			
MADRS-improvement (%)	0.554***	0.543***	0.168	0.835***	0.952***	–		
∑ ECT-sessions	−0.249	−0.162	−0.268	−0.112	−0.139	−0.081	–	
Stimulation dose (%)	0.420**	0.610***	0.051	0.346*	0.387*	0.395*	−0.216	–

GREAT, Göttingen Response to ECT Assessment Tool; MADRS, Montgomery Åsberg Depression Rating Scale; ECT, electroconvulsive therapy. Correlations: * *p* < 0.05, ** *p* < 0.01, *** *p* < 0.001. GREAT-score (0 to 14 points); gender (1 = male, 2 = female); responder (0 = non-responder, 1 = responder); MADRS Δ (pre-measurement minus post-measurement; positive values indicate higher delta); MADRS-improvement (percentage of MADRS symptom reduction from pre- to post-measurement; higher values indicate higher improvement), *N* = 45, *n* = 40 for MADRS variables.

### GREAT: ROC-analysis and cut-off value

The ROC-analysis with GREAT-score as test variable (0 to 14 points) and response as state variable (‘yes’ versus ‘no’) led to an AUC of 0.841 (asymptotic significance: *p* < 0.001; see Fig. 1(b), classification as ‘excellent discriminator’).^23^ The highest Youden-Index was 0.57 at a cut-off value of 7/14 points, leading to a sensitivity of 92%, and a false positive rate of 35% (specificity: 65%; negative predictive value: 86.7%, positive predictive value: 76.7%). A possible cut-off value at seven points was partially supported when comparing 95% CIs of GREAT-scores between responders and non-responders: for non-responders (mean 5.95), the 95% CI range was 5.06 to 6.84. For responders (mean 8.84), the 95% CI range was 7.96 to 9.72, the latter suggesting a slightly higher cut-off value. However, for 8/14 points, the Youden-Index was reduced to 0.51: an eight-point cut-off resulted in high costs on the sensitivity side (76%) to achieve a slightly smaller false positive rate (25%). In sum, a cut-off at seven points led to the best discriminatory results; it predicted the response for *n* = 36 out of 45 patients (80.0%) correctly.

**Fig. 2:**
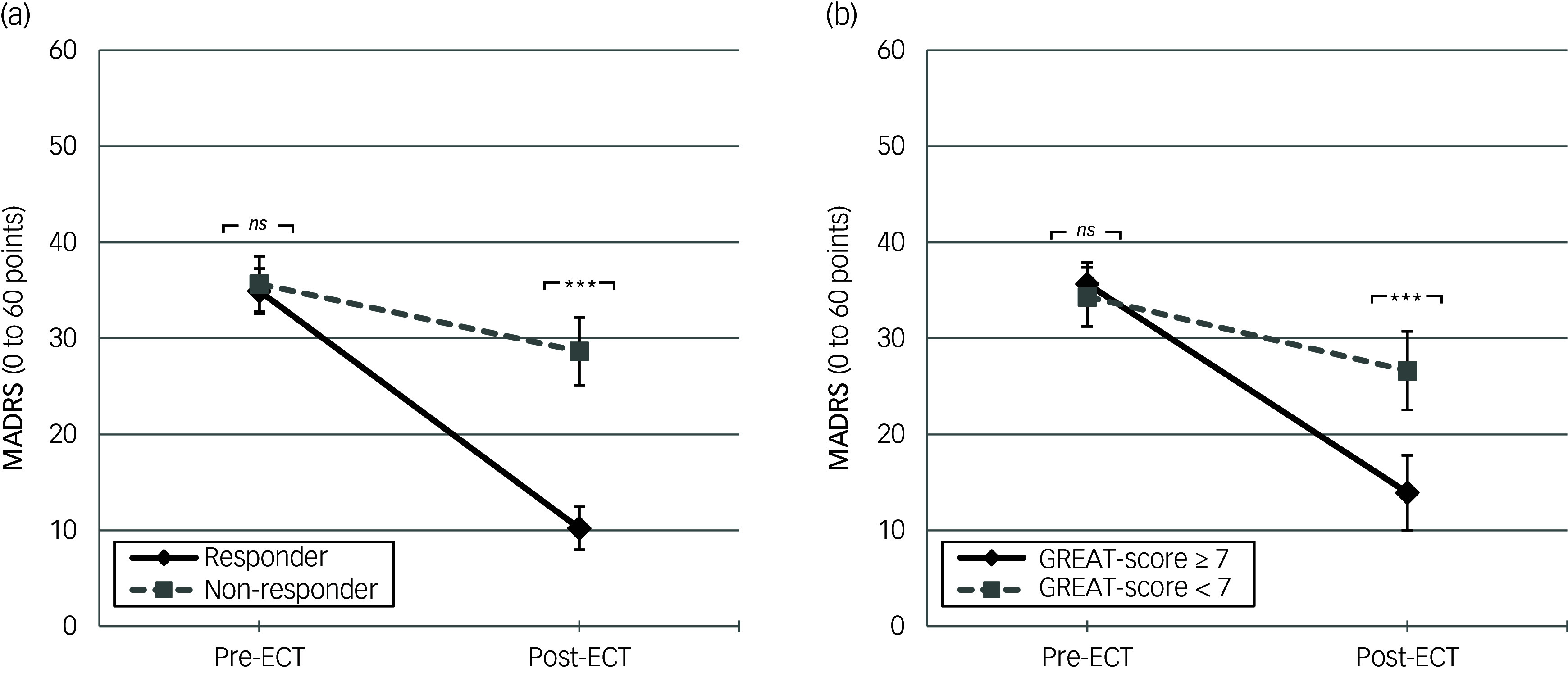
Montgomery Åsberg Depression Rating Scale (MADRS) pre–post improvement. Mean values with 95%-CIs and Bonferroni corrected pairwise comparisons of the patients’ MADRS-score (range from 0 to 60 points) pre- and post-electroconvulsive therapy (ECT)-series differentiated by (a) response post hoc (MADRS-improvement of ≥ 50%: responder: *n* = 23, non-responder: *n* = 17). (b) Göttingen Response to ECT Assessment Tool (GREAT)-score cut-off value *a priori* (GREAT-score of ≥ seven points: *n* = 27, < seven points: *n* = 13); *ns* = non-significant, *** *p* < 0.001; *n* = 40.

Two GLMs for repeated measures (within-subjects: MADRS pre- and post-measurement) were calculated to further evaluate the discriminatory ability of a possible GREAT cut-off value at seven points. First, response as defined by MADRS-improvement (*n* = 40 patients) was implemented as between-groups discriminator into the reference model (first GLM, see Fig. 2(a)). Here, a significant interaction effect for group (responder status) × repeated measurement was found (*F*(1, 38) = 52.28; *p* < 0.001, partial *η* ^2^ = 0.58). As expected for the reference model, corrected pairwise comparisons showed no significant MADRS-difference between responders and non-responders pre-ECT (*M*_*diff*_ = 0.73 points, *p* = 0.699) and a significant difference post-ECT (*M*_*diff*_ = 18.43 points, *p* < 0.001). Second, the GREAT cut-off value of seven points was implemented as between-groups discriminator into the second model, instead of responder status (second GLM, see Fig. 2(b)). Results again showed a significant interaction effect for group (GREAT cut-off value) × repeated measurement (*F*(1, 38) = 18.52, *p* < 0.001, partial *η* ^2^ = 0.33). Again, corrected pairwise comparisons showed no significant MADRS-difference pre-ECT between both groups (*M*_*diff*_ = 1.36 points, *p* = 0.497) but a significant difference post-ECT (*M*_*diff*_ = 12.69 points, *p* < 0.001). In sum, the second model led to smaller effects, but the general pattern of an increasing MADRS-difference from pre- to post-measurement between patients with < 7 points and ≥ 7 points was confirmed.

In *n* = 9 out of 45 patients (20.0%), the response was not predicted correctly by a GREAT cut-off value ≥ 7 points: (1) *n* = 2 still benefited from the treatment but did not exceed the cut-off value (MADRS-improvement: 51.5 and 55.6%); (2) *n* = 7 exceeded the cut-off value, but did not respond – still, *n* = 5 of these showed a pre-post-MADRS-improvement (between 14.6 and 45.7%).

### GREAT items and response

We correlated all GREAT items with responder status, as summarised in Table 4. Overall, items 1, 2 and 7 showed positive correlations (*p* < 0.05 to < 0.001), and distribution patterns between responders and non-responders deviated significantly from an equal distribution in the anticipated direction (*p* < 0.05 to < 0.01). For example, a higher proportion of older patients was found in the responder subgroup, while a higher proportion of younger patients occurred in the non-responder subgroup (item 1, see Table 4). For items 3 to 6, correlations did not reach significance, and distributions did not deviate significantly between both groups. Still, psychotic (item 5) and psychomotor symptoms (item 6) occurred more frequently within the responder subgroup.

**Table 4:** GREAT (Göttingen Response to ECT Assessment Tool) items and responder status

Variable	Correlation with response		GREAT-scoring			
	*r*		0	1	2	*p* ^a^
I1: Age	0.475***	Responder:	5 (20.0%)	4 (16.0%)	16 (64.0%)	*0.003* ^**^
		Non-responder:	11 (55.0%)	6 (30.0%)	3 (15.0%)	
I2: Episode duration	0.384**	Responder:	4 (16.0%)	3 (12.0%)	18 (72.0%)	0.018*
		Non-responder:	8 (40.0%)	6 (30.0%)	6 (30.0%)	
I3: Pharmacotherapy response	0.155	Responder:	18 (72.0%)	2 (8.0%)	5 (20.0%)	0.650
		Non-responder:	17 (85.0%)	1 (5.0%)	2 (10.0%)	
I4: Depression severity	−0.037	Responder:	0 (0.0%)	11 (44.0%)	14 (56.0%)	0.437
		Non-responder:	1 (5.0%)	6 (30.0%)	13 (65.0%)	
I5: Psychotic symptoms	0.241	Responder:	17 (68.0%)	2 (8.0%)	6 (24.0%)	0.231
		Non-responder:	17 (85.0%)	2 (10.0%)	1 (5.0%)	
I6: Psychomotor symptoms	0.226	Responder:	5 (20.0%)	7 (28.0%)	13 (52.0%)	0.303^b^
		Non-responder:	7 (35.0%)	7 (35.0%)	6 (30.0%)	
I7: Comorbid borderline PD	0.340*	Responder:	1 (4.0%)	0 (0.0%)	24 (96.0%)	0.045*
		Non-responder:	5 (25.0%)	1 (5.0%)	14 (70.0%)	

PD, personality disorder. Correlations: * *p* < .05, ** *p* < .01, *** *p* < .001. Please see Table 1 for all item- and scoring-formulations. Responder status: 0 = non-responder, 1 = responder.

a. *p*-values for the Freeman–Halton extension of the Fisher Exact probability test (2 × 3 matrices) instead of b. χ^2^ test, due to expected frequencies ≤ 5 in single cells for six out of seven variables; *N* = 45 (responder: *n* = 25, non-responder: *n* = 20).

## Discussion

To the best of our knowledge, this is the first study that developed and prospectively tested a clinical tool for the prediction of response to ECT in patients with depression. The GREAT was designed as a prospective, time-efficient and easy-to-use clinical application. In this study, the GREAT-score (0 to 14 points) showed a significant correlation with dichotomous ECT response (*r* = 0.585) and an explained variance of 30.7% in MADRS-improvement from pre- to post-ECT. Each point on the GREAT-score was associated with a MADRS-improvement of 7.3%. Based on the ROC analysis, our results led to an AUC of 0.841 and suggested a preliminary cut-off value of seven points, which best differentiated between individuals that responded or did not respond to ECT (sensitivity: 92%, false positive rate: 35%). Based on this cut-off value, individual ECT response was predicted correctly in 80% of cases. Eventually, analyses showed that the (*a priori* measured) GREAT-score led to significantly better MADRS-improvement for patients ≥ 7 points (*v*. < 7 points), largely reflecting the improvement pattern of (*post hoc* defined) responders versus non-responders over the course of ECT. Overall, we consider the results of our study to be very encouraging. The information included in the seven items of GREAT is available from routine clinical care, making it a potentially useful and economic tool for clinical practice.

During the last years, several studies have addressed the question of ECT response prediction in depression. Two studies used symptom-based approaches with established depression rating scales. The first study using a regression model including MADRS total score and MADRS item no. 2 (reported sadness) yielded AUCs of ≤ 0.65 for response at the end of treatment.^24^ Another study using network outcome analysis on the Hamilton Rating Scale for Depression^25^ found three baseline symptoms (retardation, hypochondriasis and suicidality) to be predictive of remission. However, neither of the two studies provided an approach for application in clinical practice. Further models have been developed in recent years using large data-sets to predict ECT response. One research group trained a Bayesian network model with predictors derived from three meta-analyses and a data-set of 248 treatment courses. This model yielded an AUC of 0.686 for the validation set for predicting remission to ECT and an AUC of 0.528 for non-response.^14^ Another group used multivariate linear regression analysis in 1892 patient data-sets to develop a prediction model for depression outcome post-ECT.^13^ The final model reached an adjusted *R* ^2^ of 19%. For the secondary outcome response, optimism-adjusted C-statistic was 0.682. Authors from Japan reported a machine learning approach in a retrospective chart review of 177 patients.^26^ Their model predicted individual patient remission (CGI-I) with 71% accuracy. It is important to note that all these models were calculated based on retrospective data-sets – prospective studies to estimate the probability of response prior to ECT do not to exist to this point.

It is important to emphasise, too, that the GREAT cut-off value of seven points should not be used to generally exclude patients with an indication for ECT from this treatment. Despite its predictive value for ‘classically defined’ responders/non-responders (see above), a symptom reduction of less than 50% can still mean a clinically relevant improvement for patients with severe or treatment-resistant depressive disorders. In this context, the concept of difficult-to-treat depression defines treatment goals beyond response and remission. Symptomatic control, functional recovery and quality of life improvement are suggested to be important components of chronic disease management.^27^ Previous studies suggested a threshold of eight points on the MADRS for clinician-rated minimal improvement and a threshold of five points as minimal important difference according to patient ratings.^28,29^ Given the mean MADRS of 34.95 points in our population, a MADRS-improvement of 22.9% would be considered clinically relevant. Therefore, the GREAT-score should currently be used exclusively to give clinicians a relevant estimate of the likelihood and the extent of the individual response to ECT.

### Limitations

There are some limitations regarding this study, which is preliminary in nature. First, the sample size clearly limited exploratory analyses concerning the relative impact of single predictors (e.g., demographic variables as ethnicity) and generation of weighted models. Second, further potential clinical predictors suggested by recent studies were not yet included in the tested GREAT version (e.g., personality disorders in general,^30,31^ presence of childhood trauma and maltreatment^32,33^ or presence of psychosocial stressors).^13,34^ Third, the instrument may be further improved by modifications: in the version used here, the rating of the GREAT items 4−6 was based on the assessment of the patients’ psychopathology ≤ 7 days before the beginning of the ECT series. However, psychotic or psychomotor symptoms that were present prior to hospitalisation may have already decreased significantly at the time of the assessment due to the application of benzodiazepines or antipsychotics. Consequently, this may have led to an underestimation of the GREAT-score for patients who showed a particularly ‘ECT-responsive’ psychopathology. The presence of these symptoms will be assessed for the current episode irrespective of a possible improvement by medication in the next version of GREAT. Fourth, regarding item no. 3 (insufficient response to pharmacotherapy), we can be criticised for not using established instruments like the Maudsley staging method (MSM)^35,36^ to capture the degree of treatment resistance. However, as the MSM itself contains items for symptom severity and episode duration, these factors would have been included twice in our instrument. Furthermore, the assessment of pharmacological treatment was limited to the current episode. In our experience it is often difficult (and sometimes impossible) to obtain a reliable medication history concerning treatments in previous episodes. Thus, we decided to use the information from the current episode exclusively as it has been applied in a previous meta-analysis.^9^

### Future development of GREAT

This prospective study shows that a seven-item assessment tool based on readily available clinical information can predict response to ECT in patients with depression with relatively good accuracy. GREAT is a time-efficient instrument with an intuitive feasibility in routine care. It does not require patient cooperation, as ratings can be based on other sources of information, such as third-party anamnesis, medical documents and observation by medical staff. A modified version of GREAT that addresses the limitations discussed above is currently in preparation. This instrument will be tested in a much larger multicentre study and hopefully lead to an even more accurate prediction of ECT outcome. Future studies may combine this clinical tool with other and multimodal (e.g., molecular, (epi-)genetic, neuroimaging) markers to ultimately provide an instrument that reliably predicts ECT outcome in the individual patient and thus can guide and facilitate clinical decision-making.

## Data Availability

The data that support the findings of this study are available from the corresponding author upon reasonable request.
